# Women and Cardiovascular Health: Unraveling Gender-Specific Factors, Risks, and Therapeutic Approaches in Contemporary Medicine

**DOI:** 10.7759/cureus.56440

**Published:** 2024-03-19

**Authors:** Zeeshan Ahmed Khattak, Anas Ahmad, Haseeb Khan, FNU Mainka, Jaisingh Rajput, Salman Khan, Abdul Momin Malik, Zahid Ali Kaimkhani, Muhammad Ahsan, Taher Janoowala

**Affiliations:** 1 Department of Pharmacy, Burjeel Medical City, Abu Dhabi, ARE; 2 Department of Pharmacy, Aman Hospital, Doha, QAT; 3 Medical Intensive Care Unit, Dar Ul Sehat Hospital, Karachi, PAK; 4 Medicine and Surgery, Shaheed Mohtarma Benazir Bhutto Medical University Larkana, Larkana, PAK; 5 Family Medicine, Montgomery Baptist Family Medicine Residency Program, Montgomery, USA; 6 Medical Unit, Divisional Headquarters Teaching Hospital/Gomal Medical College, Dera Ismail Khan, PAK; 7 Department of Internal Medicine, Jinnah Sindh Medical University, Karachi, PAK; 8 Department of Anatomy, College of Medicine, King Saud University, Riyadh, SAU; 9 Department of Internal Medicine, Jinnah Postgraduate Medical Centre, Karachi, PAK; 10 Medicine, Saifee Hospital, Karachi, PAK

**Keywords:** women's health, therapeutic approaches, risks, gender-specific factors, cardiovascular health

## Abstract

Background and objective

Cardiovascular diseases (CVDs) constitute a significant global health challenge, causing millions of deaths annually and straining healthcare systems worldwide. This study aimed to investigate and elucidate gender-specific factors, risks, and therapeutic approaches related to cardiovascular health in women within the context of contemporary medicine.

Methodology

We conducted a prospective observational study spanning one year (November 2022 to October 2023) at the Peshawar Medical Complex Hospital, to meticulously explore the field of women's cardiovascular health. With a diverse cohort of 435 women (age range: 18-55 years), representing various socioeconomic backgrounds and geographic locations, our study aimed to elicit comprehensive insights. Through structured interviews covering reproductive history, lifestyle, and psychosocial aspects, coupled with clinical assessments, we gathered multifaceted data. Statistical analysis was done using SPSS Statistics version 23.0 (IBM Corp., Armonk, NY). By employing descriptive and t-tests for quantitative analysis and by thematically analyzing qualitative insights, our approach ultimately sought to provide a nuanced understanding of gender-specific factors impacting women's cardiovascular health.

Results

The study, involving 435 women, revealed various prevalent cardiovascular risk factors. Notable findings include a high incidence of a family history of CVD (n=213, 48.96%, p=0.013), hypertension (n=207, 47.58%), hypercholesterolemia (n=114, 26.21%), elevated triglycerides (n=162, 37.24%), and diabetes (n=64, 14.71%). Physical inactivity was also significantly more common (53.56%, p=0.004) compared to those engaging in regular activity. Women-specific risk factors comprised miscarriage (n=191, 43.91%). Therapeutic preferences varied, with a majority opting for lifestyle modifications (n=263, 60.39%) and pharmacological interventions (n=331, 76.33%).

Conclusions

This study provides a comprehensive understanding of prevalent cardiovascular risk factors, distinctive women-specific contributors, and diverse therapeutic preferences, highlighting the importance of personalized and targeted interventions to optimize women's cardiovascular health outcomes in contemporary medicine.

## Introduction

Cardiovascular diseases (CVDs) constitute a pervasive global health challenge, claiming countless lives and straining healthcare systems worldwide [1]. Traditionally considered a predominantly male affliction, there has been an evolving recognition of the unique facets of cardiovascular health in women [2]. However, significant knowledge gaps persist, hindering our ability to devise targeted strategies for the prevention and treatment of CVD in women [3]. Globally, CVDs account for approximately 17.9 million deaths each year, making them the leading cause of mortality according to the World Health Organization (WHO). This oversight is particularly glaring when considering the unique physiological, hormonal, and sociocultural factors that distinctly influence cardiovascular well-being in women [4].

A crucial aspect of our investigation revolves around the exploration of gender-specific factors that intricately shape cardiovascular health in women [5]. Hormonal dynamics, specifically those associated with reproductive phases such as menstruation, pregnancy, and menopause, introduce distinctive considerations that influence cardiovascular function. For example, estrogen, a hormone with known cardio-protective effects, undergoes fluctuations during different life stages, thereby impacting women's susceptibility to CVD [6]. A nuanced understanding of these gender-specific factors is essential for designing tailored interventions that take into account the unique physiological intricacies of women [7,8].

Furthermore, this research seeks to elucidate the risks associated with CVD in women, acknowledging that while some risk factors may overlap with those in men, others are distinct and require specific attention. Factors such as socioeconomic status, lifestyle choices, and psychosocial stressors, when viewed through a gender-specific lens, contribute significantly to the nuanced understanding of cardiovascular risks in women [9]. Unraveling these distinctive risk profiles is essential for designing targeted preventive strategies and personalized interventions that align with the unique needs of female patients [10].

In the context of contemporary medicine's shift toward personalized and precision approaches, the importance of tailoring therapeutic strategies based on gender-specific considerations cannot be overstated [11,12]. In light of this, this study endeavors to explore and evaluate the efficacy of therapeutic approaches in the context of women's cardiovascular health, recognizing the need to align medical interventions with the unique physiological and pathophysiological aspects of the female cardiovascular system. In short, the study aims to investigate and elucidate gender-specific factors, risks, and therapeutic approaches related to cardiovascular health in women within the context of contemporary medicine.

## Materials and methods

Study design and setting

We adopted a prospective observational study design, spanning a period of one year from November 2022 to October 2023. The study was conducted at the Peshawar Medical Complex Hospital in Peshawar, aiming to provide comprehensive insights into women's cardiovascular health within a tertiary healthcare setting.

Sample size

A total of 435 women (age range: 18 to 55 years) were recruited from the diverse patient population at Peshawar Medical Complex Hospital. The selection criteria encompassed a spectrum of age groups, socioeconomic backgrounds, and geographic representation, ensuring a comprehensive and representative sample for robust analyses.

Inclusion and exclusion criteria

The study enrolled females aged 18-55 years, willing to provide informed consent, without prior CVDs, and open to structured interviews and assessments. All males and those women outside the age range of 18-55 years were excluded. Individuals unwilling to provide informed consent, those who were pregnant during the study period, and those with medical conditions affecting hormonal balance and any factors interfering with accurate cardiovascular assessments or active data collection were excluded.

Data collection

Data collection encompassed a multidimensional approach. Structured interviews were conducted to gather detailed information on reproductive history, hormonal dynamics, lifestyle choices, and psychosocial stressors. Clinical data, including blood pressure measurements, lipid profiles, and other relevant biomarkers, were obtained through standardized medical assessments. Qualitative insights were derived through in-depth interviews and focus group discussions to capture the nuanced experiences and perspectives of the participants.

Statistical analysis

Statistical analysis was done using SPSS Statistics version 23.0 (IBM Corp., Armonk, NY). Quantitative data underwent descriptive analysis, including the calculation of frequencies, means, and percentages. T-tests were employed to compare means and frequencies between groups where appropriate. Qualitative data were analyzed thematically to identify recurrent patterns and themes. The integration of both quantitative and qualitative findings provided a comprehensive understanding of gender-specific factors influencing women's cardiovascular health. A p-value <0.05 was considered statistically significant.

Ethical approval

﻿We obtained ethical approval from the Ethical Review Committee, Gomal Medical College, D.I. Khan (Ref. No. 211/GJMS/ER, dated August 22, 2022). The committee ensured that all research procedures adhered strictly to ethical guidelines, prioritizing confidentiality, privacy, and informed consent of the participants. A rigorous assessment of potential risks and benefits underscores the commitment to safeguarding the well-being and rights of the women participating in the study.

## Results

The study comprised 435 women with diverse demographic characteristics (Table 1). The majority fell within the age groups of 40-49 and 50-55 years, constituting 30.11% (n=131) and 37.94% (n=165), respectively. The educational backgrounds of the participants varied, with 51.96% (n=226) having a college education, followed by 27.81% (n=121) with a university degree. In terms of marital status, the majority were married (n=281; 64.59%), while 24.14% (n=105) were single. Regarding children, 47.35% had one child (n=206), and 31.27% (n=136) had none. Socioeconomic status showed a fairly even distribution, with 46.89% (n=204) belonging to the middle-income category, 32.65% (n=142) in the high-income bracket, and 20.46% (n=89) in the low-income group.

**Table 1:** Demographic Distribution of the Participants (n=435) P-value <0.05 was considered statistically significant

Characteristic	Frequency (n=435)	Percentage	t	p
Age group (years)				
18-29	47	10.81	4.27	0.023
30-39	92	21.14		
40-49	131	30.11		
50-55	165	37.94		
Education				
School	88	20.23	3.48	0.073
College	226	51.96		
University	121	27.81		
Marital Status				
Single	105	24.14	1.79	0.17
Married	281	64.59		
Divorced	33	7.59		
Widowed	16	3.68		
Offspring				
0	136	31.27	2.66	0.76
1	206	47.35		
2	78	17.94		
3 or more	15	3.44		
Socioeconomic Status				
Low Income	89	20.46	4.36	0.049
Middle Income	204	46.89		
High Income	142	32.65		

As shown in Table 1, there was a statistically significant difference (p=0.023) in age between the groups. A positive t-value (4.27) indicates that the mean age in one group was likely higher than the other. There was also a statistically significant difference (p=0.073) in educational attainment between the groups. A positive t-value (3.48) suggests that one group had, on average, more years of education than the other. No statistically significant difference (p=0.170) was found regarding marital status between the groups. This means that the distribution of marital statuses (single, married, divorced, widow) was likely similar across the groups. There was no statistically significant difference (p=0.76) in the number of offspring between the groups. This suggests the distribution of the number of children was likely similar across the groups. There was a statistically significant difference (p=0.049) in socioeconomic status between the groups. A positive t-value (4.36) indicates that one group had, on average, a higher socioeconomic status than the other.

Among 435 participants, a family history of cardiovascular disease emerged as a prominent risk factor (n=213, 48.96%; Table 2). This prevalence was significantly higher compared to those without a family history (p=0.013). Physical activity levels also showed a significant difference, with 233 participants (53.56%) reporting a sedentary lifestyle compared to those engaging in occasional or regular activity (p=0.004). While hypertension (n=207, 47.58%) and overweight (n=162, 37.24%) were prevalent, other risk factors like smoking (n=6, 1.37%), alcohol consumption (three occasional drinkers, 0 regular drinkers), diabetes (n=64, 14.71%), hypercholesterolemia (n=114, 26.21%), and anxiety (n=29, 6.67%) did not exhibit significant differences across groups (Table 2). This analysis suggests that family history and physical inactivity are key risk factors requiring targeted interventions in this population.

**Table 2:** Distribution of Cardiovascular Risk Factors Among the Participants (n=435) P-value <0.05 was considered statistically significant CVD: Cardiovascular Disease

Risk Factor	Frequency (n)	Percentage	t	p
Family History of CVD	213	48.96	48.33	0.013
Smoking Habit				
Smoker	6	1.37	2	0.295
Past Smoker	2	0.45		
Alcohol Consumption				
Occasional Drinker	3	0.68	1	0.5
Regular Drinker	0	0		
Physical Activity				
Sedentary Lifestyle	233	53.56	4.23	0.004
Occasional Physical Activity	11	2.54		
Regular Physical Activity	191	43.9		
Hypertension	207	47.58		
Diabetes	64	14.71		
Overweight	162	37.24		
Hypercholesterolemia	114	26.21		
Anxiety	29	6.67		

The study revealed significant women-specific cardiovascular risk factors among the participants (Table 3). Among those, early or premature menopause emerged as a potential concern, affecting 56 women (12.87%). Although not statistically significant at the conventional p<0.05 level (p=0.065), this trend warrants further investigation. Other factors like menopausal hormone therapy (n=46, 10.57%), polycystic ovary syndrome (n=19, 4.37%), preterm delivery (n=14, 3.21%), miscarriage (n=191, 43.91%), eclampsia (n=9, 2.07%), gestational hypertension (n=28, 6.44%), and gestational diabetes (n=12, 2.76%) did not show significant differences in prevalence, suggesting that those may not be major contributors to cardiovascular risk in this population based on the current analysis.

**Table 3:** Prevalence of Women-Specific Cardiovascular Risk Factors in the Study Population (n=435) P-value <0.05 was considered statistically significant

Women-Specific Cardiovascular Risk Factors	Frequency (n)	Percentage	t	p
Early or Premature Menopause	56	12.87	2.187	0.065
Menopausal Hormone Replacement Therapy	46	10.57		
Polycystic Ovary Syndrome	19	4.37		
Preterm Delivery	14	3.21		
Miscarriage	191	43.91		
Eclampsia	9	2.07		
Gestational Hypertension	28	6.44		
Gestational Diabetes	12	2.76		

Table 4 provides a detailed overview of the cardiovascular health profile and risk assessment of the participants. The table includes mean values with standard deviations and ranges for key biomarkers such as blood pressure, cholesterol levels, and inflammatory markers. Each biomarker is accompanied by clinical interpretations and risk assessments, offering insights into the prevalence of hypertension, hypercholesterolemia, elevated triglyceride levels, and diabetes within the study population.

**Table 4:** Comprehensive Cardiovascular Health Profile and Risk Assessment BP: Blood Pressure; SD: Standard Deviation; LDL: Low-Density Lipoprotein; HDL: High-Density Lipoprotein; CRP: C-Reactive Protein; NT-proBNP: N-Terminal Pro-B-Type Natriuretic Peptide

Biomarker	Mean ± SD	Range	Clinical Interpretation	Risk Assessment
Systolic BP (mmHg)	125.4 ± 12.8	90-150	Normal: <120, elevated: 120-129, hypertension stage 1: 130-139, hypertension stage 2: ≥140	47.58% prevalence of hypertension
Diastolic BP (mmHg)	80.2 ± 8.5	60-100	Normal: <80, elevated: 80-89, hypertension stage 1: 90-99, hypertension stage 2: ≥100	
Total Cholesterol	195.6 ± 25.3	150-250	Desirable: <200, borderline high: 200-239, high: ≥240	26.21% exhibit hypercholesterolemia
LDL Cholesterol	120.8 ± 18.6	80-160	Optimal: <100, near-optimal: 100-129, borderline high: 130-159, high: 160-189, very high: ≥190	
HDL Cholesterol	50.3 ± 6.2	40-70	Low: <40, better: 40-59, best: ≥60	
Triglycerides	130.2 ± 30.5	90-200	Normal: <150, borderline high: 150-199, high: ≥200	37.24% have elevated triglyceride levels
Blood Glucose	95.7 ± 10.2	70-120	Normal fasting: <100, prediabetes: 100-125, diabetes: ≥126	14.71% have diabetes
C-Reactive Protein	3.2 ± 1.5	1-6	Normal: <1, moderate Risk: 1-3, high risk: >3	3.2 ± 1.5 mg/L indicates low to moderate risk
Homocysteine	9.8 ± 2.7	6-15	Normal: 5-15 µmol/L	9.8 ± 2.7 µmol/L within normal range
NT-proBNP	45.6 ± 8.3	30-60	Normal: <125 pg/mL	45.6 ± 8.3 pg/mL indicates normal levels

Table 5 outlines various therapeutic approaches in the study population. The majority (60.39%) opted for lifestyle modifications, focusing on behavioral changes, diet, and physical activity, with an emphasis on long-term adherence. Pharmacological interventions, chosen by 76.33%, involved medication regimens guided by standard protocols, focusing on adherence and side effect management. Behavioral therapy, selected by 20.77%, centered on psychological aspects, with active participation in therapy sessions for mental health improvement. Surgical interventions (5.80%) involve invasive procedures, requiring informed decision-making for surgeries and postoperative care. Integrative medicine, chosen by 14.01%, integrated holistic approaches with conventional treatments, fostering openness to alternative therapies. Personalized/precision medicine (29.95%) tailored treatments based on individual factors, incorporating genetic data for informed participation. Nutritional counseling (38.65%) emphasized dietary adherence and lifestyle changes for cardiovascular health. Physical rehabilitation (16.91%) involved structured exercise programs, coordinating with healthcare teams for functional improvement. Mindfulness practices (27.54%) integrated mind-body techniques for stress reduction and mental well-being. Telemedicine interventions (12.56%) offered remote healthcare services with technological integration, enhancing accessibility and patient satisfaction.

**Table 5:** Therapeutic Approaches in Contemporary Medicine

Therapeutic Approach	Frequency	Percentage	Primary Focus	Clinical Integration	Patient Engagement	Efficacy and Adherence Factors
Lifestyle Modification	125	60.39	Behavioral changes, diet, physical activity	Integrated into patient education and counseling	Active participation in lifestyle changes	Long-term adherence, lifestyle impact
Pharmacological Interventions	158	76.33	Medication regimens, drug therapy	Standard protocols guided by medical professionals	Medication adherence and management	Side effect management, dosage compliance
Behavioral Therapy	43	20.77	Psychological aspects, stress management	Collaborative approach with mental health professionals	Participation in therapy sessions	Mental health improvement, coping mechanisms
Surgical Interventions	12	5.8	Invasive procedures, surgical interventions	Coordinated efforts with surgical and medical teams	Informed decision-making for surgeries	Post-surgery recovery, complications awareness
Integrative Medicine	29	14.01	Holistic approaches, complementary therapies	Combined with conventional treatments for synergy	Openness to alternative therapies	Holistic well-being, integration with mainstream
Personalized/Precision Medicine	62	29.95	Tailored treatments based on individual factors	Incorporation of genetic and personalized data	Informed participation in personalized plans	Treatment response, individualized outcomes
Nutritional Counseling	80	38.65	Dietary guidance, nutritional education	Integrated into comprehensive patient care plans	Dietary adherence and lifestyle changes	Nutrient balance, impact on cardiovascular health
Physical Rehabilitation	35	16.91	Structured exercise programs, rehabilitation	Coordination with physical therapists and healthcare teams	Active involvement in rehabilitation plans	Functional improvement, physical activity levels
Mindfulness Practices	57	27.54	Mind-body techniques, stress reduction	Integrated into behavioral and mental health strategies	Regular practice of mindfulness techniques	Stress reduction, mental well-being
Telemedicine Interventions	26	12.56	Remote healthcare services, virtual consultations	Technological integration for remote patient monitoring	Engagement in virtual health consultations	Accessibility, patient satisfaction

## Discussion

This study, conducted over one year and involving 435 women from diverse backgrounds, aimed to explore gender-specific factors, risks, and therapeutic approaches related to cardiovascular health. The demographic distribution revealed a broad representation across various age groups, educational backgrounds, marital statuses, and socioeconomic statuses, contributing to the robustness and generalizability of the findings. The prevalence of family history of CVD among 48.96% (n=213) of participants highlights the importance of genetic factors in shaping cardiovascular health, aligning with established literature on familial predisposition as a significant risk factor [13]. Additionally, in terms of physical activity patterns, a high prevalence of sedentary behavior 53.56% (n=233) is a serious concern, as physical inactivity is a well-established risk factor for CVD. Encouraging regular physical activity should be a priority in cardiovascular health interventions for women, aligning with the findings of previous studies emphasizing the benefits of exercise in preventing CVD [14,15].

The high prevalence rates of hypertension (47.58%, n=207), overweight (37.24%, n=162), hypercholesterolemia (26.21%, n=114), and diabetes (14.71%, n=64) highlight a significant health concern. These findings closely parallel global trends, emphasizing the mounting burden of non-communicable diseases, particularly in low- and middle-income countries. The observed results seamlessly align with existing literature, reinforcing the established connection between these health conditions and an elevated cardiovascular risk [16,17]. Women-specific cardiovascular risk factors, such as miscarriage (43.91%; n=191) and early or premature menopause (12.87%; n=56) were prevalent in the study population. These findings highlight the importance of considering reproductive factors in cardiovascular risk assessment, consistent with existing literature emphasizing the impact of hormonal changes on heart health in women [18,19].

The therapeutic approaches chosen by the participants reveal a preference for pharmacological interventions (76.33%, n=158), lifestyle modification (60.39%, n=125), nutritional counseling (38.65%, n=80), personalized/precision medicine (29.95%, n=62), mindfulness practices (27.54%, n=57), and behavioral therapy (20.77%; n=43). This aligns with the contemporary shift toward personalized and patient-centric care in cardiovascular medicine [20]. The inclusion of telemedicine interventions (12.56%, n=26) reflects an evolving landscape of holistic and technologically integrated approaches in cardiovascular care [21].

Limitations of the study

One limitation of this study is its single-center design (conducted exclusively at the Peshawar Medical Complex Hospital), which may limit the generalizability of the findings to the broader population.

## Conclusions

The prevalence of hypertension, hypercholesterolemia, obesity, and diabetes among the participants underscores the need for personalized lifestyle interventions. The detailed health profile and risk assessment offer valuable insights for targeted interventions. Participants' preference for lifestyle modifications, pharmacological interventions, and personalized medicine aligns with the evolving paradigm of patient-centric cardiovascular care. Integrating these findings into clinical practice can enhance preventive strategies and optimize therapeutic outcomes, contributing to the advancement of women's cardiovascular health.
